# Oldest known captive Saker Falcon (*Falcocherrugcherrug*) at 31 years old

**DOI:** 10.3897/BDJ.13.e159703

**Published:** 2025-08-08

**Authors:** Rusko Petrov, Dariya Cholakova

**Affiliations:** 1 Trakia University, Stara Zagora, Bulgaria, Bulgaria Trakia University Stara Zagora, Bulgaria Bulgaria; 2 Green Balkans - Stara Zagora NGO, Stara Zagora, Bulgaria Green Balkans - Stara Zagora NGO Stara Zagora Bulgaria; 3 Freelance ecologist, Stara Zagora, Bulgaria Freelance ecologist Stara Zagora Bulgaria

**Keywords:** raptor conservation, captive breeding, Bulgaria, falcons lifespan

## Abstract

Saker Falcons play an important role in the ecosystems they are part of, but their population has been globally declining. Despite many Saker Falcons being bred in captivity, both for falconry and conservation, little is known about the longevity of the species. We report a Saker Falcon has lived to the unprecedented age for the species - 31 years old, at the Green Balkans Wildlife Rehabilitation and Breeding Centre in Stara Zagora, Bulgaria, highlighting the importance of careful management and expert care. To our knowledge, this is the oldest living Saker Falcon, suggesting that, with a better understanding of the species needs and better care, Saker Falcons can live longer than previously believed. Longer lifespans of Saker Falcons in captivity could improve conservation efforts and result in greater success for Saker Falcon conservation projects.

## Introduction

The Saker Falcon (*Falcocherrug*) has been most recently categorised as Endangered in 2021 by the IUCN (International 2021) due to very rapid population decline. In the Bulgarian Red Data Book, it is categorised as Critically Endangered (Domuschiev et al. 2011). The greatest threats for the species are the degradation of essential prey habitat, illegal and unsustainable capture, electrocution on power lines, habitat loss and the use of chemicals in agriculture (International 2021). The global population is estimated to be between ca. 6,100-14,900 pairs (Dixon 2007, Dixon 2009, Kovács et al. 2014). Between 2002-2021, the population has seen a 44.6% decline (based on median estimates), with a minimum-maximum decline of 12-71%. Due to the uncertainty of the estimates used, the population decline trend has been placed in the band for a 50-79% decline over three generations. The European population is relatively small and has been estimated to be around 430-630 breeding pairs (International 2021). Only 15-16 breeding pairs were observed in Czechia, 31-32 breeding pairs in Slovenia and 214-230 breeding pairs in Hungary during 2008 (Ragyov et al. 2009). In Bulgaria, a national Saker Falcon survey was conducted in 2002-2004 and found no breeding pairs (Ragyov et al. 2014). The decline of the Saker Falcon in Europe is due to prey habitat loss, intentional and unintentional poisoning, electrocution on power lines, human disturbance, a decline in sheep pastoralism, direct persecution, infrastructure development, nest robbing, agriculture intensification and unsustainable capture of wild birds for falconry (Kovács et al. 2014, Ragyov et al. 2014, Spasov et al. 2017, Petrov et al. 2021).

In response to the declining population of the Saker Falcon, several conservation projects have been established. In Bulgaria, this included captive breeding and releasing: in order to restore the local breeding population, a re-introduction programme was initiated in 2011 at the Wildlife Rehabilitation and Breeding Centre (WRBC), part of Green Balkans - Stara Zagora NGO (Lazarova et al. 2021). The programme included captive breeding, different rearing techniques and release through the hacking method (Sherrod et al. 1987). The released birds were found to be successfully breeding in the wild and, in 2018, the first Saker Falcon chicks hatched for the first time in 20 years (Dixon et al. 2020, Lazarova et al. 2021). Studies on the lifespan of Saker Falcons are limited, particularly concerning wild birds. Information and official records for the longevity of birds in the wild are hard to come by due to the fact that it is difficult to track birds from hatching to old age and it is very rare for an animal to die from natural causes in the wild. The greatest mortality occurs in young inexperienced birds, while, for adults the probability of death remains roughly the same each year after successfully rearing young (Klimkiewicz and Futcher 1989). Available data suggest they typically live between 5 to 7 years in the wild (Dixon et al. 2020), with a maximum recorded lifespan of 18 years and 8 months (Fransson et al. 2023). However, birds in the wild rarely die from old age, so records for longevity mainly come from birds that live in captivity. To the best of our knowledge, there is no scientifically documented record of the maximum lifespan of the Saker Falcon in captivity. In captivity, animals are well cared for, they receive medical treatment and regular health checkups, daily meals and protection from predators and competitors. This allows them to live out their full natural lifespan and die from old age, which helps establish the maximum lifespan of different species. For instance, captive Canada Geese have been known to live for up to 33 years in captivity (Johnsgard 1968), whereas, in the wild, Canada Geese have been recorded to live for up to 23 years (Klimkiewicz and Futcher 1989).

At the WRBC in Bulgaria, where a conservation project for the restoration of the Saker Falcon is taking place, a male Saker Falcon named Romeo has reached the remarkable age of 31 years old - making him the oldest known Saker Falcon in captivity and marking the longest official lifespan record for the species. This exceptional case presents an interesting and notable deviation from the expected lifespan of the species and prompts a closer look at the factors that might have contributed to such longevity.

## Material and methods

At the WRBC, male and female Saker Falcons were paired for breeding and their offspring were released as part of the species re-introduction programme in Bulgaria. The specifications of the Saker Falcon aviaries, rearing and feeding methodology were previously described in detail by Petrov et al. (2021) and Petrov et al. (2022). At WRBC, many Saker Falcons have been part of the said project, but no other has lived to such an impressive age as the Saker Falcon, Romeo. The oldest age that the other Saker Falcons from the breeding group have lived to is 25 years old. Romeo was part of the conservation breeding project at the WRBC for 7 years before he was “retired”. His life at the conservation centre started when he was imported into Bulgaria in March 2012 from Bojnice Zoo, Slovakia. His medical record showed he had hatched on 02.05.1994, proving he turned 31 years in 2025. In 2012, Romeo was paired with a female named Juliet. The pair was part of the breeding project for 7 years - the first breeding season of Romeo and Juliet was in 2012 and the last was in 2018. Romeo was breeding until he was 24 years old, but, at 25 years old, he stopped breeding most likely due to his old age.

## Results

For the period Romeo and Juliet were paired, they laid 35 eggs, from which 21 chicks were successfully reared - three were left in the centre to take part in the breeding project and 18 were released into the wild in Bulgaria. The details of each breeding season and reproductive success are summarised in Table 1.

## Discussion

The case of Romeo, the oldest known Saker Falcon in captivity at the remarkable age of 31 years old, offers a unique opportunity for understanding and studying Saker Falcon longevity, health and, in general, provides an opportunity for a better understanding of management practices for raptors in captivity. While data about the lifes of wild Saker Falcons are limited, studies on other falcon species show falcons face numerous challenges that shorten their lives in the wild, including food shortages (Gangoso et al. 2020, Foysal and Panter 2024), environmental hazards, such as pesticides (Castagna et al. 2024) and diseases (Radcliffe et al. 2024). In addition to these dangers, wild falcons also face a range of other threats, including poisoning, persecution and electrocution. (Ragyov et al. 2014, Dixon et al. 2020). Furthermore, migration is dangerous and taxing, with species that migrate or disperse prior to breeding age facing even more threats than non-migratory species (Strandberg et al. 2009). In contrast, Saker Falcons in captivity benefit from a controlled environment with regular and secure meals, veterinary care, shelter, access to water and protection from predators and threats. Romeo's incredible lifespan is a testament to the expert care he has received, as well as our growing understanding of falcons nutritional needs and advanced veterinary care. These factors contribute to better overall health and allow Saker Falcons and birds of prey in general to live far longer in captivity than in the wild. With the development of tailored diets, husbandry and health monitoring, Saker Falcons in captivity might be able to live even longer than previously thought.

To our knowledge, Romeo's age is the first known record of such longevity for the species, both in captivity and in the wild. No other Saker Falcon is known to have reached such an age. Proving the limit of the Saker Falcon lifespan in captivity is still unknown. However not all Saker Falcons in captivity can reach such an age. Unique factors that might have contributed to Romeo's long life may include his genetic makeup, high tolerance to stress and lack of previous health conditions. Nevertheless, Romeo's extraordinary age highlights the potential for increased longevity in captive birds of prey when expert care practices are employed and shows that there is a need for further research into the factors that contribute to the maximum age span of birds of prey in captivity, in order for more raptors in captivity to reach such an impressive age and live out their full lifespan.

## Conclusions

In conclusion, it is a great achievement for conservation that an individual from a globally threatened species such as the Saker Falcon has lived to 31 years old. It provides valuable insight into the species ecology, physiology and morphology, emphasising the possibility for an extended life of an important species. By better understanding the conditions that have contributed to such a long life, future conservation efforts could be more successful. With expert care, Saker Falcons can survive for longer than previously believed, showcasing the importance of a carefully managed environment and a tailored diet. Romeo's age serves as further proof that, with the right care, there is hope for the preservation and long-term survival of this species.

## Figures and Tables

**Table 1.** Breeding success of the Saker Falcon pair, Romeo and Juliet, including information about the fate and sex of the chicks.

Year	Eggs	Fertile	Dead	Left for breeding	Released	Sex
2012	4	0	0	0	0	♂ - 0, ♀ - 0
2013	5	3	1	1	1	♂ - 0, ♀ - 1
2014	5	5	2	1	2	♂ - 2, ♀ - 0
2015	5	5	0	0	5	♂ - 1, ♀ - 4
2016	6	6	0	1	5	♂ - 2, ♀ - 3
2017	5	5	1	0	4	♂ - 2, ♀ - 2
2018	5	5	4	0	1	♂ - 0, ♀ - 1

## References

[B12907706] Castagna Fabio, Montano Luigi, Lombardi Renato, Pagano Angelo, Gigliotti Andrea, Bava Roberto, Lupia Carmine, Costagliola Anna, Giordano Antonio, Palma Ernesto, Britti Domenico, Liguori Giovanna (2024). Understanding Environmental Contamination Through the Lens of the Peregrine Falcon (*Falcoperegrinus*). Environments.

[B12907818] Dixon A (2007). Saker Falcon breeding population estimates, Part 1: Europe. Falco.

[B12907827] Dixon A (2009). Saker Falcon breeding population estimates, Part 2: Asia. Falco.

[B12907723] Dixon Andrew, Ragyov Dimitar, Izquierdo David, Weeks Darren, Rahman Md. Lutfor, Klisurov Ivaylo (2020). Movement and Survival of Captive-Bred Saker Falcons *Falcocherrug* Released by Wild Hacking: Implications for Reintroduction Management. Acta Ornithologica.

[B12913717] Domuschiev D., Stoyanov G., Michev T., Petrov C., Vatev I., Ruskov K., Golemanski V. (2011). Red Data Book of Republic Bulgaria.

[B12907734] Foysal Mohammod, Panter Connor T. (2024). Synergistic effects of climate and urbanisation on the diet of a globally near threatened subtropical falcon. Ecology and Evolution.

[B12913782] Fransson T., Kolehmainen T., Kroon C., Jansson L., Wenninger T. EURING list of longevity records for European birds. https://euring.org/data-and-codes/longevity-list-2023.

[B12907743] Gangoso Laura, Viana Duarte S., Dokter Adriaan M., Shamoun‐Baranes Judy, Figuerola Jordi, Barbosa Sergio A., Bouten Willem (2020). Cascading effects of climate variability on the breeding success of an edge population of an apex predator. Journal of Animal Ecology.

[B12907810] International BirdLife (2021). Falco cherrug. IUCN Red List of Threatened Species. 2021: e.T22696495A204182473.

[B12913791] Klimkiewicz M. K., Futcher A. (1989). G.Longevity records of North American birds: Supplement 1. Journal of Field Ornithology.

[B12913800] Kovács A., Williams N., Galbraith C. (2014). Saker Falcon, *Falco cherrug* Global Action Plan (SakerGAP).

[B12907755] Lazarova Ivanka, Petrov Rusko, Andonova Yana, Klisurov Ivaylo, Dixon Andrew (2021). Re-introduction of the Saker Falcon (*Falcocherrug*) in Bulgaria - preliminary results from the ongoing establishment phase by 2020. Biodiversity Data Journal.

[B12907774] Petrov R., Andonova Y., Gancheva Y., Klisurov I. (2021). Implications of captive breeding for the reintroduction of the Saker falcon (*Falcocherrug*) in Bulgaria. Agricultural Science and Technology.

[B12907765] Petrov R., Andonova Y., Yarkov D., Dicheva A. (2022). Diets for captive breeding and hacking of saker falcons (*Falcocherrug*) in Bulgaria. Agricultural Science and Technology.

[B12907783] Radcliffe Robin W., Booms Travis L., Henderson Michael T., Barger Chris P., Bowman Dwight D., Lucio-Foster Araceli, Virapin Manigandan L., Dhondt Keila V., Levitskiy Alexander A., Reinoso-Perez Maria Teresa, Ito Mio, Anderson David L., Nielsen Ólafur K. (2024). Gyrfalcon disease ecology: A survey across western Alaska. Journal of Raptor Research.

[B12913877] Ragyov D., Kmetova E., Dixon A., Franz K., Koshev Y., Nedialkov N. (2009). Saker Falcon (*Falcocherrug*) Reintroduction in Bulgaria: Feasibility Study.

[B12913864] Ragyov D., Biserkov V., Gradev G., Ivanov I., Stoynov E., Stoyanov G., Domuschiev D., Dixon A. (2014). Past and present status of the Saker Falcon, *Falcocherrug* (Aves: Falconidae) in Bulgaria. Acta Zoologica Bulgarica.

[B12913916] Sherrod S. K., Heinrich W. R., Burnham W. A., Barclay J. H., Cade T. J. (1987). Hacking: A method for releasing peregrine falcons and other birds of prey.

[B12913925] Spasov S., Hristov I., Eaton M., Nikolov S. C. (2017). Population trends of common birds in Bulgaria: Is their status improving after the EU accession?. Acta Zoologica Bulgarica.

[B12907801] Strandberg Roine, Klaassen Raymond H. G., Hake Mikael, Alerstam Thomas (2009). How hazardous is the Sahara Desert crossing for migratory birds? Indications from satellite tracking of raptors. Biology Letters.

